# Support Vector Machine model for hERG inhibitory activities based on the integrated hERG database using descriptor selection by NSGA-II

**DOI:** 10.1038/s41598-019-47536-3

**Published:** 2019-08-21

**Authors:** Keiji Ogura, Tomohiro Sato, Hitomi Yuki, Teruki Honma

**Affiliations:** RIKEN Center for Life Science Technologies, 1-7-22 Suehiro-cho, Tsurumi-ku, Yokohama 230-0045 Japan

**Keywords:** Drug safety, Machine learning, Statistical methods

## Abstract

Assessing the hERG liability in the early stages of drug discovery programs is important. The recent increase of hERG-related information in public databases enabled various successful applications of machine learning techniques to predict hERG inhibition. However, most of these researches constructed the datasets from only one database, limiting the predictability and scope of the models. In this study, a hERG classification model was constructed using the largest dataset for hERG inhibition built by integrating multiple databases. The integrated dataset consisted of more than 291,000 structurally diverse compounds derived from ChEMBL, GOSTAR, PubChem, and hERGCentral. The prediction model was built by support vector machine (SVM) with descriptor selection based on Non-dominated Sorting Genetic Algorithm-II (NSGA-II) to optimize the descriptor set for maximum prediction performance with the minimal number of descriptors. The SVM classification model using 72 selected descriptors and ECFP_4 structural fingerprints recorded kappa statistics of 0.733 and accuracy of 0.984 for the test set, substantially outperforming the prediction performance of the current commercial applications for hERG prediction. Finally, the applicability domain of the prediction model was assessed based on the molecular similarity between the training set and test set compounds.

## Introduction

Drug safety is an important issue in pharmaceutical research and development (R&D)^1^. The major reasons for failures in clinical trials in the 2000s were lack of efficacy and safety (approximately 30%)^2^. Cardiotoxicity, hepatotoxicity, genotoxicity, and phototoxicity are frequently observed toxicities. There are 30 to 40% reports of cardiotoxicity and hepatotoxicity in clinical trials and post-approval studies, respectively^3^. The human Ether-a-go-go Related Gene potassium channel (hERG) is a critical contributor to drug-induced prolongation of the QT interval and arrhythmia, called Torsades de Pointes (TdP)^4,5^. Since hERG binds and is inhibited by structurally diverse compounds, hERG is regarded as a major anti-target for drug discovery. Several drugs (*e*.*g*., terfenadine^6^, cisapride^7^, and sertindole^8^) have been withdrawn from the market because of the inhibition of hERG. Consequently, the assessment of hERG inhibition is essential in drug discovery projects. A patch clamp assay and a cardiotoxicity assay using iPS cell-derived cardiomyocytes have been developed to monitor the effects on hERG^9^, and are commonly used to assess cardiotoxicity at later stages in drug discovery. However, if the cardiotoxicity of a compound is revealed at a later stage, it would severely impact the project. Thus, the evaluation of the hERG blockade of a compound at an early stage is quite important in the drug discovery process. In early drug discovery stages such as screening and hit to lead optimization, performing costly and time-consuming assays is difficult; therefore, the development of an *in silico* model to predict hERG inhibition would be useful. Many *in silico* models have been developed by both structure and ligand-based approaches^10,11^. These studies included statistical models based on the 2D or 3D structures of small compounds, and structure-based approaches employing docking simulations using a modeled 3D structure of hERG. Although the electronic microscopy structure of hERG was reported in 2017^12^, docking simulations with hERG are still difficult challenges, due to its high flexibility. However, recent increases in the bioactivity information about hERG inhibitors in public databases (*e*.*g*., ChEMBL, PubChem) have accelerated the improvement of statistical models using machine learning techniques

The previously reported machine learning models of hERG inhibition were summarized by Wang^10^ and Villoutreix^11^. As machine learning methods, PLS, Bayesian, and Neural Networks were mainly used in the early 2000s, and subsequently Random Forest (RF) and Support Vector Machine (SVM) have been often used since 2010. In recent studies about classification models, Czodrowski^13^ constructed RF models using descriptors calculated by Rdkit^14^, based on 3,721 compounds measured in a binding assay and 765 compounds measured in a functional assay collected from ChEMBL^15^. The prediction models were constructed separately from each data set, and showed prediction accuracies of 0.797–0.801 and 0.692–0.907, respectively. Shen *et al*. used SVM combined with 4D-fingerprints and traditional 2D and 3D VolSurflike molecular descriptors to build a model based on the PubChem hERG Bioassay dataset, including 876 compounds^16^. The SVM model achieved an accuracy of 0.87 for the test set of 456 compounds. Wang *et al*. developed hERG classification models using naïve Bayesian classification and recursive partitioning based on molecular properties and the ECFP_8 fingerprints, and recorded 85% accuracy for test set^17^. The group also combined pharmacophore modeling and machine learning techniques (naïve Bayesian classification and SVM) and recorded 82.1% accuracy for the external test set^18^. Liu *et al*. developed the Bayesian classification model using four molecular properties (MW, PSA, AlogP, and pKa_basic), as well as extended-connectivity fingerprints (ECFP_4), based on a dataset of 2,644 compounds including compounds tested on hERG in the literature and FDA-approved drugs, divided into a training set of 2,389 compounds and a test set of 255 compounds^19^. In addition, further validation was performed experimentally using an external data set of 60 compounds by Doddareddy^20^. The model showed an accuracy of 0.91 for the test set and 0.58 for the external test set. In 2015, one of the most recent models, Pred-hERG, was reported by Braga *et al*.^21^. To build the classification models, Morgan fingerprints and Chemistry Development Kit descriptors were calculated for 5,984 compounds in ChEMBL using RDKit and the PaDEL descriptor plugin for KNIME, respectively. The consensus model built by each descriptor showed the best performance (Correct Classification Rate = 0.84), and is freely available at http://labmol.com.br/predherg/. Schyman *et al*. combined 3D similarity conformation and 2D similarity ensemble approach, and achieved 69% sensitivity and 95% specificity on an independent external data set^22^.

**Figure 1:**
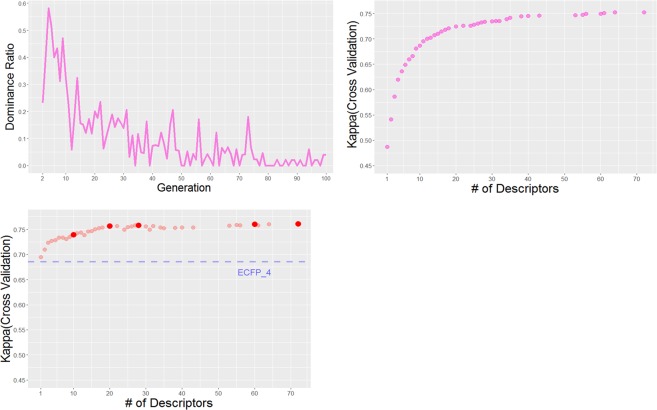
The result of descriptor selection by NSGA-II. (**a**) Ratio of dominated Pareto solutions of previous generation at generation *t*. (**b**) The Kappa statistics and the number of used descriptors of the descriptor sets in the 100th generation. (**c**) Results of the combination of the Pareto solutions descriptor set and ECFP_4. The selected descriptor sets for model building are highlighted. The kappa statistics of the SVM model only using ECFP_4 is shown as dashed line.

Despite the successful applications of the increased hERG information, most of the current researches constructed their datasets from only one database, because the differences in the data format and ontology made the integration of various databases difficult. Thus, the dataset sizes used to construct hERG prediction models in most of the previous studies were limited to less than 3,000, as reported by Wang^10^ (Fig. 1). Our previous research^23^ integrated the hERG-associated information from ChEMBL^15^, GOSTAR^24^, NIH Chemical Genomics Center dataset registered in PubChem^25^, and hERGCentral^26^ into a dataset consisting of more than 291,000 structurally diverse compounds. The analysis revealed that the hERG-related entries derived from the respective databases showed different distributions for various molecular properties, according to the information source of each database (journals, patents, HTS library, etc.). Thus, the construction of a prediction model using a single database could result in limited prediction performance and applicability.

In this study, we developed a novel hERG classification model by SVM, using the most comprehensive hERG blocker information from the integrated database. To avoid overfitting of the prediction model, the descriptor set was optimized to show high accuracy with a small number of descriptors by a genetic algorithm, to find the Pareto optimal for multiple objective functions. Subsequently, the importance of each of the selected descriptors for hERG prediction was assessed, according to the results of the descriptor selection. Finally, model validation and comparisons of the prediction performance with commercially available hERG prediction models were conducted. Its applicability domain was also evaluated based on the molecular similarity to the training compounds.

## Methods

### Data set

The integrated dataset for hERG inhibitory activity^23^, consisting of ChEMBL, GOSTAR, the NIH Chemical Genomics Center (NCGC) dataset in PubChem bioassay, and hERGCentral, was used to build a prediction model. As reported by Brag *et al*.^27^, assay entries derived from literatures often contains unreliable values and duplicates. Assay entries for which data validity problems or potential duplicates were indicated in ChEMBL were removed. The references of the assay entries were also collated not to derive same data from multiple database. Then, the compounds in the four databases were merged into a dataset of 9,890 hERG inhibitors (IC_50_ ≤ 10 μM or ≥50% inhibition at 10 μM) and 281,329 inactive compounds (IC_50_ > 10 μM or <50% inhibition at 10 μM) according to their standardized chemical structures (Table 1). For compounds with contradictory assay results, the compound was assigned as an inhibiter or inactive when more than two thirds of the reports agreed to either class. IC_50_ data were prioritized in the assignment to percent inhibition data when both values were available. This dataset is available in Supplementary Information. Detailed procedures of the curation to format ontologies, the standardization of chemical structures, and the classification of inhibitors/inactives, were reported in the previous study^23^. The raw assay values are available at our home page (http://drugdesign.riken.jp/hERGdb/) except the data from GOSTAR, a commercial database. The dataset was randomly split into the training set (70%, 6,923 inhibitors/196,918 inactives) and the test set (30%, 2,966 inhibitors/84,395 inactives) for external validation. For genetic algorithm-based descriptor selection, another training set with fewer inactives was prepared by clustering the inactive compounds by ECFP_4 to reduce the calculation time. From each of the 6,923 clusters, the cluster center was selected to create a trimmed training set containing 6,923 molecules for both hERG inhibitors and inactive compounds.

**Table 1:** Details of the integrated dataset.

Database	hERG inhibitors	Inactive compounds
ChEMBL (version 22)	4,793	5,275
GOSTAR	3,260	3,509
NCGC	232	1,234
hERGCentral	4,321	274,536
hERG integrated dataset	9,890	281,329

### Machine learning algorithms

Prior to the detailed optimization including descriptor selection and parameter tuning, discrimination models using linear discrimination, single-layer neural network, deep neural network, random forest, and SVM with ECFP_4 were built using Pipeline Pilot with the default parameter settings for preliminary assessment. Dividing the reduced training set containing even numbers of hERG inhibitors and inactive compounds into 70% and 30% for preliminary training and validation, prediction performances of the discrimination models were evaluated as shown in Table 2. Among the algorithms, SVM recorded the highest kappa statistics among the machine learning models for validation (0.64, 0.794, 0.643, 0.858, and 0.863, respectively). Despite the recent reports of various successful applications of deep neural network, the deep neural network model underperformed SVM and random forest in this study. Since the network was built only using the default setting of Pipeline Pilot, consisting of two hidden layer with 50 nodes and dropout of 0.25 probability using R package “deepnet”, intense optimization of hyperparameters would be necessary to obtain a deep neural network model outperforming other machine learning algorithms. While random forest also showed the equivalent prediction performance to the SVM model, SVM was selected for the model building with descriptor selection and further optimization because of the slight advantage in Kappa statistics and possibility of parameter tunings such as cost factor and gamma value for the RBF kernel function.

**Table 2:** Preliminary assessment of machine learning algorithms.

Algorithm	TP/FN FP/TN	Accuracy	Sensitivity	Specificity	BAC*	Kappa	ROC
Linear discrimination	946/226 199/989	0.820	0.807	0.832	0.820	0.640	0.890
Neural network	919/253 232/956	0.794	0.784	0.805	0.794	0.589	0.867
Deep neural network	1076/96 333/855	0.818	0.918	0.720	0.819	0.637	0.907
Random forest	986/186 150/1038	0.858	0.841	0.874	0.858	0.715	0.927
SVM	996/176 147/1041	0.863	0.850	0.876	0.863	0.726	0.925

^*^BAC: balanced accuracy.

### Molecular descriptors

To construct a hERG prediction model, 424 descriptors and ECFP_4 were calculated, using MOE^28^ and Pipeline Pilot^29^. Using MOE, 192 2D descriptors and 134 3D descriptors were computed. Using Pipeline Pilot, 98 descriptors and the ECFP_4 structural fingerprint were computed. To calculate the 3D descriptors, 3D molecular conformers were generated and minimized by a AMBER10:EHT force field^30^ with solvent reaction field using MOE. The full list of the descriptors is available in the Supporting Information (Table S1).

### Descriptor selection

Using too many descriptors for machine learning often results in an over-complicated model that could lead to overfitting. Thus, descriptor selection based on the Non-dominated Sorting Genetic Algorithm-II (NSGA-II)^31^ was applied to maximize the prediction performance with the minimal number of descriptors.

NSGA-II is an optimization algorithm to find the Pareto optimal for multiple objective functions. First, an initial population is generated randomly. The individuals in each population are sorted according to their dominance levels. The individuals in the Pareto front, which no other individuals shows higher values for all of the objective functions than, are defined as level 1. Then, the individuals only dominated (overtaken for all of the objective functions) by the level 1 individuals are defined as level 2, and so on. In addition to the dominance level, the crowding distance is also calculated to rank the individuals with the same dominance level. The crowding distance represents how close an individual is to its neighbors. In each generation, a given number of individuals is selected, based on their dominance level. If individuals with the same dominance level remain, then those with a larger crowding distance are selected. The selected individuals become the parents of the next generation, and generate the offspring by mutation and crossover. The selected individuals and offspring compose the population of the next generation. This procedure is iterated until it reaches the specified number.

In this study, NSGA-II was employed to simultaneously optimize both the number of descriptors and the prediction performance by the SVM model. To reduce the computing cost due to the high-dimensionality, descriptor selection was performed using 424 descriptors, and then the combination of the obtained descriptor sets and ECFP_4 was tested. Before the descriptor selection, three preprocesses were performed to delete some descriptors: (1) some obviously non-relevant descriptors (*e*.*g*., the number of unconstrained chiral centers); (2) descriptors with a variance of 0; and (3) descriptors showing correlation coefficient higher than 0.85 to the other descriptors. As a result, 213 descriptors were selected for NSGA-II. The subset of the 213 descriptors was treated as an individual for NSGA-II. For each descriptor set, a SVM model to predict the hERG blocking activity was built, using the training set. The average kappa statistics of the 5-fold cross validation (kappa CV) and the number of used descriptors were defined as the objective functions for NSGA-II. The parameters of NSGA-II were defined as follows: generations = 100, population size = 50, mutation rates = 0.02 (off to on) and 0.2 (on to off), and crossover rate = 0.6. After 100 iterations, the descriptor sets in the Pareto front were combined with ECFP_4 to build final SVM models using full training set.

### Support vector machine

SVM models nonlinearly discriminate two classes of compounds, by mapping the data vectors to a very high-dimensional descriptor space and finding the hyperplane that separates the two classes with the largest margin. In this study, a radial basis function was chosen as a kernel function. During the descriptor selection, the gamma for the RBF and C, the constant for the slacks variant, were fixed to the default values of SVMlight package^32^ (1.0 and 1.0/nx, respectively (nx indicates the number of descriptors)), to reduce computational cost. After the descriptor selection, C and Gamma were optimized by a grid search in the 5-fold cross validation on the full-training set. The search ranges of C and gamma are as follows: C = 0.5, 1.0, or 2.0, Gamma = 0.5/nx, 1.0/nx, or 2.0/nx. Due to the imbalanced number of positive and negative compounds, the class weight option, which imposes a heavier penalty for errors in the minority class, was used. The weight was defined as the ratio of the positive and negative compounds in the training set. Other parameters were set to the default values of Pipeline Pilot.

### Evaluation of prediction performance

To evaluate the model performance, the following metrics were calculated.

1 $$accuracy=\frac{{\rm{TP}}+{\rm{TN}}}{{\rm{TP}}+{\rm{FN}}+{\rm{TN}}+{\rm{FP}}}$$

2 $$sensitivity=\frac{{\rm{TP}}}{{\rm{TP}}+{\rm{FN}}}$$

3 $$specificity=\frac{{\rm{TN}}}{{\rm{TN}}+{\rm{FP}}}$$

4 $$balanced\,accuracy=\frac{sensitivity+specificity}{2}$$

TP (true positives) and FN (false negatives) denote the numbers of known inhibitors predicted to be active and inactive. TN (true negatives) and FP (false positives) are the numbers of known inactives predicted to be inactive and active. Cohen’s kappa was also applied for model evaluation. Kappa measures the agreement between the predicted and observed classes and compare the agreement to that expected by chance. Kappa is defined in the following Eq. (5),

5 $${\rm{Cohen}}\mbox{'}{\rm{s}}\,kappa=\frac{{\rm{Po}}-{\rm{Pe}}}{1-{\rm{Pe}}}$$

6 $${\rm{Pe}}=\frac{({\rm{TP}}+{\rm{FN}})({\rm{TP}}+{\rm{FP}})+({\rm{TN}}+{\rm{FP}})({\rm{TN}}+{\rm{FN}})}{\#\,{\rm{of}}\,{\rm{compound}}\,\ast \,\#\,{\rm{of}}\,{\rm{compound}}}$$

where Po is the relative observed agreement and Pe is the random chance of agreement. Furthermore, the performance of the SVM model was also measured by the ROC score, defined as the area under the receiver operation curve, which plots the ratio of true positives on the axis of false positive fractions and ranges from 0 to 1.

### Comparison with commercial prediction models

The constructed SVM model was compared to three commercially available models (ACD/Percepta^33^, ADMET Predictor^34^, StarDrop^35^). ADMET Predictor and StarDrop predict the hERG pIC_50_ values of the given compounds. Thus, the predicted pIC_50_ value of 5.0 was defined as the criterion to classify positive and negative compounds. ACD/Percepta calculates the probability that a compound inhibits hERG with a K_i_ value lower than 10 μM. The predicted probability of 0.5 was defined as the threshold to classify the positive and negative compounds. Compounds that could not be evaluated by the commercial software were removed from the test set to compare the prediction performances.

### Assessment of applicability domain

To evaluate the applicability domain of the SVM model for reliable prediction, the relationship between the structural similarity and the prediction accuracy was assessed, based on the concept that the prediction of a compound similar to those in the training set could be reliable. The similarity-based approach was applied because the data distribution of the hERG dataset was quite high-dimensional and sparse due to ECFP_4 fingerprint, and difficult to estimate the data distribution in probabilistic approaches to assess the applicability domain. For each test set compound, the ECFP_4 Tanimoto similarities were calculated to all of the training compounds, and the highest similarity was employed as the metric for prediction difficulty.

## Results

### Descriptor selection

The result of the descriptor selection by NSGA-II is shown in Fig. 1. The dominance ratio describes the ratio of the Pareto solutions of generation i-1 dominated by the Pareto solutions of generation i. After the 80th generation, the dominance ratio is less than 0.1, meaning that most of the Pareto solutions have not been updated (Fig. 1(a)). Thus, 100 generations would be sufficient to optimize the two objective metrics. Among the 40 Pareto solutions in the 100th generation, the descriptor set with 35 descriptors recorded well balanced prediction performance (Accuracy = 0.870, Kappa = 0.741). With more descriptors, the kappa CV only showed a slight improvement over those using 35 descriptors (Fig. 1(b)).

The descriptor sets of the Pareto solutions in the 100th generation were combined with ECFP_4. As compared to ECFP_4 alone, the combination of descriptor sets and ECFP_4 recorded improved kappa statistics (Fig. 1(c)). As in the case of SVM models using only descriptors, the predictive performance slightly improved when more descriptors were combined with ECFP_4. Then, a further study was performed to confirm the balance between the number of descriptors and the prediction performance. From the Pareto solutions combined with ECFP_4, a descriptor set showing the highest kappa CV value was chosen from the range of every 10 descriptors. By eliminating the descriptor sets showing lower kappa CV values than those using fewer descriptors, five descriptor sets (10, 20, 28, 60, and 72 descriptors highlighted in Fig. 1(c)) were selected for model construction using the whole training data. The statistics of the cross validation by the prediction models constructed by the five descriptor sets are shown in Table 3. All models showed sufficient prediction performances. Among the five models, the model with 72 descriptors yielded the best performance (accuracy = 0.983, balanced accuracy = 0.854, Kappa = 0.735), and the selected descriptors seemed to be consistent with the features of hERG inhibitors reported in a previous study^36^.

**Table 3:** Statistical results of 5-fold cross validation for promising Pareto models.

# of descriptors	TP/FN FP/TN	Accuracy	Sensitivity	Specificity	BAC	Kappa
10	4,706/2,217 1,390/195,528	0.982	0.680	0.993	0.836	0.714
20	4,816/2,107 1,373/195,545	0.983	0.696	0.993	0.844	0.725
28	4,721/2,102 1,374/195,544	0.983	0.696	0.993	0.845	0.726
60	4,926/1,997 1,413/195,505	0.983	0.712	0.993	0.852	0.734
72	4,949/1,974 1,430/195,488	0.983	0.715	0.993	0.854	0.735

The accuracy and the kappa CV of the model combining ECFP_4 and all the calculated 424 descriptors were 0.901 and 0.714, while those of the combination of ECFP_4 with 212 descriptors, the largest descriptor set evaluated in the descriptor selection by NSGA-II, were 0.983 and 0.728, respectively. From the comparison between these two models, the model by descriptor selection showed slightly higher predictive performance with the smaller number of descriptors. This result indicated that the complexity of the model was reduced by removing redundant and non-relevant descriptors. Therefore, the SVM model based on ECFP_4 and 72 descriptors was selected as the descriptor set with a good balance between the number of descriptors and the predictive performance. The 72 selected descriptors are listed in Supplementary Table S2.

### Assessment of selected descriptors

**Figure 2:**
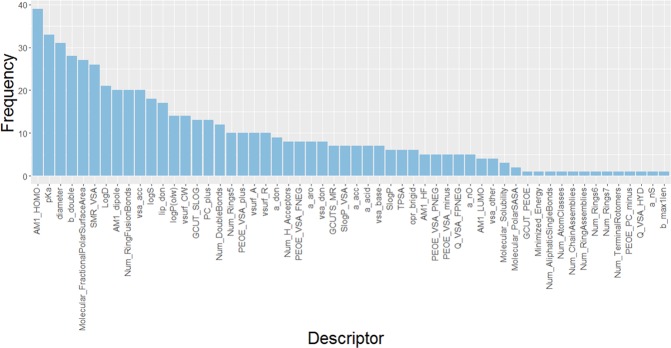
Frequency of descriptors in the 100th generation of the Pareto solutions.

The importance of each molecular descriptor was evaluated by the number of occurrences in the 40 Pareto solutions in the last generation. Frequently used molecular descriptors can be considered as important descriptors for hERG inhibition. The frequency of each descriptor is shown in Fig. 2.

According to analyses of site-directed mutagenesis and homology modeling^37–39^, Tyr652 and Phe656 were identified as the important residues forming electrostatic interactions (cation-π) and π stacking interactions with several known hERG inhibitors. The most frequently selected descriptor is AM1_HOMO, which could represent the electrostatic conditions in the aromatic ring, and correlates the π-π interaction with Phe656. The second one is pKa, which could represent the cation-π interaction with Tyr652. In fact, many hERG inhibitors have an aromatic ring at the terminal position of the ligand and a basic amine that is easily protonated at physiological pH. In our previous study, while about 80% of the inactive compounds had no positively charged atoms, more than half of the hERG inhibitors contained at least one positively charged atom. Therefore, atomic charge-related descriptors, such as PEOE_VSA_plus, GCUT_PEOE, and PC_plus, were also chosen in the descriptor selection.

In addition to the atomic charge, several previous studies reported that hERG liability compounds tend to be larger, more hydrophobic, more flexible, and have fewer H-bond acceptors^36,40^. The molecular size was expressed by the diameter, and the molecular refractivity-based descriptors SMR_VSA, and SMR_VSA were employed in our models. The hydrophobic feature was expressed by logP-based descriptors such as logD, logP(o/w), and SlogP_VSA. The hydrophobicity is also related to the polar surface area, and thus Molecular_Fractional_PolarSurfaceArea was also selected. These descriptors might be rationalized by the hydrophobic nature of the pore region of hERG, due to the high number of hydrophobic amino acids. Thus, the potency generally increases with the logP of the ligand.

The molecular flexibility was represented by Num_Aliphatic SingleBonds, b_bond, Num_Doublebond, and opr_brigid which show the rigidity of molecules. Two descriptors related to the number of double bonds were included. Num_DoubleBonds counts the number of all double bonds and b_double counts the number of double bonds excluding those in aromatic rings. Most of the ligands that showed potent activity for hERG have nitrogen-containing alkyl chains^41^, and the flexibility might be considered as an important feature of hERG inhibition.

Some pharmacophore models^42^ and a mutational analysis suggested that hydrogen bonds with Thr623, Ser624 and Val625 play an important role to inhibit hERG. The features about the hydrogen bond acceptors (vsa_acc, a_acc and Num_H_Acceptors) were selected by NSGA-II. Overall, these frequently selected descriptors seemed to be consistent with the features of hERG inhibitor reported in previous studies.

### Integration of the databases

**Figure 3:**
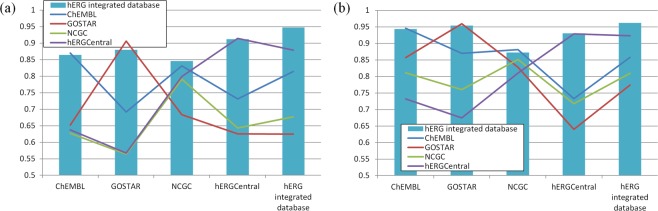
The ROC scores of the SVM models built from the hERG integrated database (light blue bar), ChEMBL (blue line), GOSTAR (red line), NCGC (green line), and hERGCentral (purple line), using (**a**) ECFP4, (**b**) ECFP4 and 72 descriptors as the explanatory variables. The horizontal axis corresponds to the data source of the test set.

Because of the heterogeneity of the assay protocols for hERG inhibition, building a prediction model using multiple databases could harm the data consistency and possibly bring the risk of prediction performance degradation. Thus, the adequacy to employ the integrated hERG dataset to build a prediction model was assessed. To investigate the relationship between the prediction performance and the data sources, the training set and the test set were separated by the original database of each assay entry to compile the data sets corresponding to the individual databases. Then, SVM model building and the prediction performance evaluation based on ROC scores were performed for all combinations of the training set and the test set. The comparison were performed for SVM models using ECFP4 to investigate the effect of data integration (Fig. 3(a)), and SVM models using ECFP4 with 72 descriptors selected by NSGA-II (Fig. 3(b)) to assess those after the descriptor selection. As previously reported^23^, the compounds in ChEMBL and GOSTAR, compiled from literatures and patents, had the different molecular property distribution and the ratio of hERG inhibitors to inactive compounds, compared to the compounds in NCGC and hERGCentral, compiled from the HTS results of chemical libraries. As the result, the SVM models built from the individual databases failed to achieve high prediction performances for test set compiled from the different databases.

For the SVM models using ECFP4, the average of ROC scores were 0.694 when the model was trained by the training set derived from the different database to the test set, and 0.870 when the training set and the test set were derived from the same database. Since hERGCentral was compiled from the HTS of nearly 300,000 compounds in the National Institutes of Health Molecular Library Small Molecule Repository, the corresponding test set could be the best approximation of the actual HTS situation. For the test set build from hERGCentral, the ROC scores of the SVM models built by ChEMBL, GOSTAR, and NCGC were all below 0.75. Since the numbers of hERG inactive compounds in ChEMBL, GOSTAR, and NCGC were far less than those in hERGCentral (Table 1), these three prediction models could not deal with structurally diverse compounds in hERGCentral dataset. The ROC scores of the SVM model built from the integrated hERG dataset showed the almost equivalent ROC scores to those by SVM models build from the corresponding training sets (0.864 for ChEMBL, 0.880 for GOSTAR, 0.846 for NCGC, and 0.912 for hERG Central), indicating that the integration of the heterogeneous assay entries caused little adverse effect in the prediction performance, and provided the better coverage of chemical space for the improved applicability.

In the aspect of the descriptor selection using NSGA-II, the ROC scores were consistently improved by the addition of 72 descriptors compared to the SVM models using only ECFP_4 for all combinations of the training sets and the test sets, indicating that the descriptor selection could successfully obtained the essential properties of hERG inhibitors in the various databases. The detailed data of the ROC scores were available in Supporting Information (Table S3) along with the corresponding Kappa statistics.

### Prediction performance of the constructed SVM model compared with commercial software

A test set of 87,361 molecules was used to validate the constructed SVM model and the commercial applications. The prediction performances for the test set are shown in Table 4. The SVM model achieved kappa statistics of 0.733 with an accuracy of 0.984, a sensitivity of 0.670 and a specificity of 0.995. The value of the kappa statistics was equivalent to that of the cross validation (0.735). These results indicated the robustness of our model against overfitting.

**Table 4:** Statistics of the SVM-model and commercial models for the test set.

Name	TP/FN FP/TN	Accuracy	Sensitivity	Specificity	BAC	Kappa	ROC
SVM-72 model*	1,987/979 410/83,985	0.984	0.670	0.995	0.833	0.733	0.962
	1,838/854 327/74,043	0.985	0.683	0.996	0.839	0.749	0.966
ACD/Percepta	1,890/802 6,518/67,852	0.905	0.702	0.912	0.807	0.304	0.890
ADMET Predictor	2,329/363 25,495/48,875	0.664	0.865	0.657	0.761	0.095	0.866
StarDrop	2,357/335 32,963/41,407	0.568	0.876	0.557	0.716	0.063	0.831

^*^For the SVM model, the first row represents the results for all test set compounds, and the second row represents the results for 77,062 compounds used with all three commercial software programs to predict the hERG inhibitory activity.

**Figure 4:**
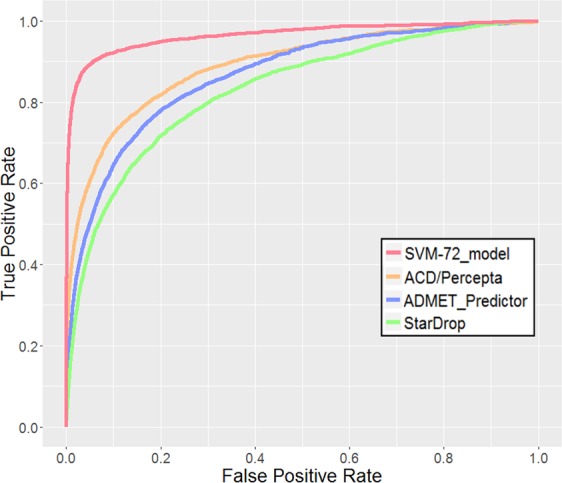
ROC curve of the SVM model, using the 72 descriptors and ECFP_4 (red), as compared to ACD/Percepta (orange), ADMET Predictor (blue), and StarDrop (green).

The prediction performance was then compared with those obtained by commercial models (ACD/Percepta, ADMET Predictor, and StarDrop). For the comparison, the compounds that cannot be predicted by some commercial models were removed from the test set, and all models were validated with 77,062 compounds. The results are shown in Table 4. Among the three commercial models, ACD/Percepta showed the best predictive performance, with kappa statistics of 0.304, an accuracy of 0.905, a sensitivity of 0.702, and a specificity of 0.912. ADMET Predictor and StarDrop recorded poor kappa statistics of less than 0.1. In Table 4, the area under the ROC curve (ROC_AUC) was also provided as another metric to assess the classification performance. The ROC_AUC value does not depend on the classification threshold, and it can evaluate the ranking ability of the classifier. To visualize the quality of the ranking, the ROC curve is shown in Fig. 4. In the evaluation of ROC_AUC, the commercial models showed good results (0.831–0.890), indicating that all of the commercial software worked well in the inhibitory potency ranking. Considering that both ADMET Predictor and StarDrop also recorded moderately high ROC scores of 0.866 and 0.831, the low kappa statistics of these two regression models did not suit the discrimination use at the criterion of IC_50_ = 10 μM. In the comparison with the constructed model, our model showed the highest ROC_AUC of 0.966, with a kappa value of 0.749 and an accuracy of 0.985. Our model clearly outperformed the commercial models. Relatively low sensitivity of the model could be caused by the use of hERGCentral data. As shown in Table 1, HTS results in hERGCentral contain huge number of inactive compounds, and thus, dramatically decreased the ratio of positive samples in the dataset. This imbalanced number of positive and negative examples could shift the threshold of the model towards negative prediction. Nevertheless, the higher ROC curve in Fig. 4 indicated that the SVM model discriminated hERG inhibitors clearly more effective than the commercial models, and the balance of positive and negative prediction could be calibrated by the setting of the threshold value when needed. The integration of the multiple databases greatly increased the hERG inhibition information, which contributed to the improved prediction performance. In particular, our model showed higher precision (0.849), as compared with commercial models (0.066–0.225). The precision is the rate that a compound predicted to be positive is actually a hERG inhibitor. The commercial software tended to predict more false positives indicating overestimation of the hERG risk of inactive compounds. Among the commercial software, ACD Percepta showed higher specificity to ADMET Predictor and StarDrop. Since ADMET Predictor and StarDrop were built to provide regression model for hERG K_i_ prediction, their dataset consisted compounds for which their binding affinity to hERG could be quantified, meaning inactive compounds showing no hERG inhibitory activity at all could not be included, and possibly resulting in lower prediction accuracy for inactive compounds. The results indicated the effectiveness of discrimination models to screen initial HTS results which distribute in broader chemical space. The regression models should be considered in the later stage where the hERG inhibitory activity of a certain hit compound should be modified through chemical synthesis. Our model would be useful in the early stage of a drug discovery program, such as HTS triage, when the purpose is to remove the compounds with a high possibility of hERG inhibition.

**Figure 5:**
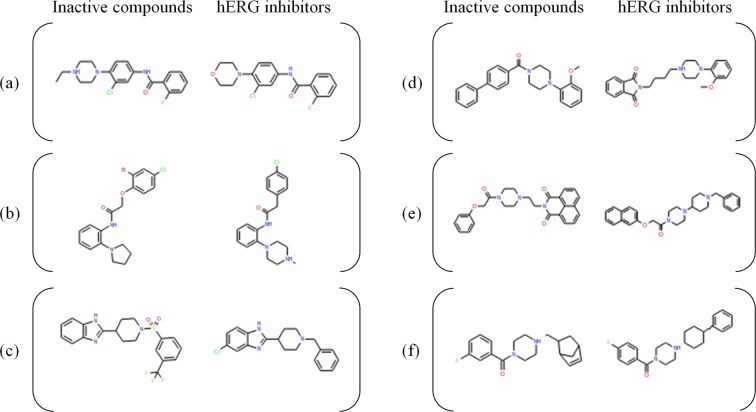
Compounds for which only the SVM model correctly predicted the activities, and their most similar hERG inhibitors. Each structure was ionized at pH7.4.

Since the commercial models show low specificity, 4,298 inactive compounds were wrongly classified as hERG inhibitors by all three commercial models. Among the 4,298 false positive cases, 4,162 compounds were correctly predicted as inactives by our SVM model. The majority of these false positive compounds had an aliphatic nitrogen atom at the center of the molecular structure. Although it is well known that π-interactions by positively charged atoms are important for hERG binding, the interactions between the nitrogen atoms contained in the misclassified false positive compounds and hERG seem to be difficult for the following reasons: (1) the influence of surrounding bulky substituents around the nitrogen atom, and (2) the nitrogen atom is generally not positively charged due to attached electron-withdrawing groups. In addition, inactive compounds that share the same scaffold with hERG inhibitors, but lack a key nitrogen atom or an aromatic ring, were also observed in the false positives. Figure 5 shows typical examples of the inactive compounds that only our SVM model correctly predicted, along with their most similar hERG inhibitors. Our SVM model successfully predicted all the inhibitor/inactive pairs, while none of the three commercial software programs could distinguish the inactive compounds. In the cases of Fig. 5(a,b), the positively charged nitrogen atoms in hERG were modified in the inactive compounds. Figure 5(c,d) represent the cases in which modifications changed the ionization tendency of the nitrogen atoms in the piperazine rings. In Fig. 5(e), the bulky chemical group could make steric clash with hERG. In Fig. 5(f), the disappearance of a terminal aromatic ring could weaken the binding affinity with hERG. The presence of two aromatic rings next to a positively charged nitrogen atom is one of the well-known pharmacophores for hERG binding, and the aromatic rings are thought to form π-electron interactions with Tyr652 and Phe654 of hERG. In the commercial models, the hERG inhibitory activity of compounds with such inaccessible or uncharged nitrogen atoms tended to be overestimated. The successful discrimination by our SVM model regarding the differences of hERG inhibition by such detailed structural changes could be benefitted by an increase of the training data in the integrated dataset. This improved prediction specificity would be especially useful in a case where structural modification to avoid hERG inhibition is needed in the hit to lead optimization process.

### Applicability domain

**Figure 6:**
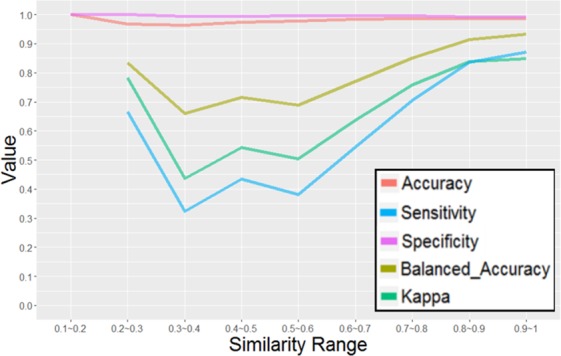
Performance metrics for the test set in each similarity range. The horizontal axis denotes similarity range, and the vertical axis indicates the values for accuracy (red), Balanced Accuracy (ocher), Kappa (green), Sensitivity (blue), and Specificity (green).

To assess the applicability domain of the classification model for reliable prediction, the relationship between the similarity of the test compounds against the training compounds and the prediction performance was investigated. While the median value of the closest Tanimoto similarities of the test set compounds to their closest training compounds was 0.726, more than half of the test compounds had structurally similar compounds in the training set. As described in Fig. 6, the compounds with high similarity to the training set showed higher prediction accuracy. However, a decrease of sensitivity and an increase in false negative compounds were observed for the compounds with lower similarities, except in the range of similarity values from 0.1 to 0.2. Since the number of compounds with similarity values of 0.1–0.3 was relatively small and contained only 9 hERG inhibitors and 86 inactive compounds, the increase of the prediction performance observed in the low similarity region did not seem to be statistically significant. These results provided insight about the applicability domain of the model for the reliable prediction. Although the specificity was not affected by the decrease of the Tanimoto similarity, the sensitivity fell below 0.5 when the Tanimoto similarity was lower than 0.6, resulting in low reliability for negative predictions. Considering the previous reports suggesting that compounds with a similarity value of 0.6 or higher tend to show similar activities^43,44^, the threshold value of 0.6 for the applicability domain criteria was further investigated. By defining the test compounds with similarities lower than 0.6 as outside of the applicability domain, 12,519 compounds among the 87,361 test set compounds were classified as outside the applicability domain. While the kappa statistics for the compounds outside of the applicability domain were still fairly high (0.512), significant degradation from those within the applicability domain (kappa statistics = 0.762) was observed. The low sensitivity of 0.392 for the outside compounds suggested that the model tends to provide negative predictions for compounds that are not similar to any compounds in the training set, containing the potential risk for false negatives. Even so, since the previously reported analysis of the integrated database revealed its higher structural diversity, covering 18.2% of the Murcko scaffolds found in the ChEMBL database, and containing hERG inhibitors with more than twice as many Murcko scaffolds as the other databases^23^, our model is expected to cover substantial chemical space to enable accurate prediction for diverse drug-like compounds, including newly designed ones. The detailed data was available at Supporting Information Table S4.

## Discussion

In this study, a novel hERG classification model based on SVM and descriptor selection by NSGA-II was developed using the integrated dataset. The amount of hERG-related information in publicly available databases has increased in recent years, and we previously integrated the hERG-associated bioactivity information from various databases. The integrated dataset consisted of more than 290,000 structurally diverse compounds covering broader chemical space, and enabled the construction of a more accurate classification model with a wider applicability domain. To avoid overfitting of the model, the descriptor set was optimized to show high accuracy with a small number of descriptors by NSGA-II. As a result of the descriptor selection, 72 descriptors were chosen to achieve a good balance between the prediction performance and the number of descriptors. The selected descriptors seemed to be reasonable and consistent with the previously reported features of hERG inhibitors. The importance of each molecular descriptor for hERG inhibition was then evaluated. The high frequencies of AM1_HOMO and pKa in the Pareto solutions were associated with the π-electron interactions with hERG. According to the analyses of site-directed mutagenesis and homology modeling, Tyr652 and Phe656 were identified as the important residues forming cation-π interactions and π stacking interactions with several known hERG inhibitors. Other frequently chosen descriptors were diameter, Molecular_FractionalPolarSurfaceArea, SMR_VSA, and LogD. To calculate the 3D molecular descriptors, the 3D conformations optimized by AMBER10:EHT force field with solvent reaction field were used in this study. Since a cryo-EM structure of hERG was reported by Wang *et al*.^12^, prediction of the binding conformation of hERG inhibitors using structure-based methods could improve the prediction in future work. The SVM model combining 72 descriptors and ECFP_4 was then developed, and achieved highly predictive performances for both the cross validation of the training set and the test set. The prediction performance was compared with three commercial models. All models were validated with 77,062 compounds, and our model clearly outperformed the three commercial models.

In addition, the applicability domain of our model was evaluated, based on the molecular similarity to the training compounds. Unsurprisingly, the compounds with high similarity to the training sets showed higher prediction accuracy, and the compounds with lower similarity decreased the sensitivity and increased the false positive ratio. By defining the similarity threshold for the applicability domain of the model as 0.6, the kappa statistics for the subset outside of the applicability domain decreased as compared to the subset within the domain (0.762 for inside the applicability domain, compared to 0.512 for outside). Despite the increased number of false negatives, our model still kept a relatively high kappa value even for the subset outside of the applicability domain according to the current definition. Considering the recent increase of hERG-associated information, the problem in the applicability domain could be gradually resolved by future updates. The model is expected to be useful to avoid hERG inhibition at the early stages of drug discovery programs and will be released publicly, along with the integrated database, at our homepage (http://drugdesign.riken.jp/hERGdb).

## Supplementary information

Supplementary information

Dataset 1
