# Supplementary material for: Support Vector Machine model for hERG inhibitory activities based on the integrated hERG database using descriptor selection by NSGA-II
Source: Sci Rep. 2019 Aug 21;9:12220. doi: 10.1038/s41598-019-47536-3 (PMC6704061; doi:10.1038/s41598-019-47536-3)
Supplement: Supplementary file 1 — Supplementary information [file 41598_2019_47536_MOESM1_ESM.docx]

Support Vector Machine model for hERG inhibitory activities based on the integrated hERG database using descriptor selection by NSGA-II

Keiji Ogura^†^, Tomohiro Sato^†^, Hitomi Yuki^†^, and Teruki Honma^†*^

RIKEN Center for Life Science Technologies, 1-7-22 Suehiro-cho, Tsurumi-ku, Yokohama 230-0045, Japan

*Corresponding author address: RIKEN Systems and Structural Biology Center, 1-7-22 Suehiro-cho, Tsurumi-ku, Yokohama 230-0045, Japan. Telephone: +81-45-503-9551, Fax: +81-45-503-9432, e-mail: honma.teruki@riken.jp

^†^Center for Life Science Technologies, RIKEN

**Table S1.** List of 424 molecular descriptors calculated for this study.

| Category | Descriptor |
| --- | --- |
| MOE 2D descriptors | apol,bpol,density,FCharge,mr,SMR,Weight,logP(o/w),logS,mutagenic,reactive,rsynth,SlogP,TPSA,vdw_area,vdw_vol,SlogP_VSA0,SlogP_VSA1,SlogP_VSA2,SlogP_VSA3,SlogP_VSA4,SlogP_VSA5,SlogP_VSA6,SlogP_VSA7,SlogP_VSA8,SlogP_VSA9,SMR_VSA0,SMR_VSA1,SMR_VSA2,SMR_VSA3,SMR_VSA4,SMR_VSA5,SMR_VSA6,SMR_VSA7,a_aro,a_count,a_heavy,a_IC,a_ICM,a_nB,a_nBr,a_nC,a_nCl,a_nF,a_nH,a_nI,a_nN,a_nO,a_nP,a_nS,b_1rotN,b_1rotR,b_ar,b_count,b_double,b_heavy,b_rotN,b_rotR,b_single,b_triple,chiral,chiral_u,lip_acc,lip_don,lip_druglike,lip_violation,nmol,opr_brigid,opr_leadlike,opr_nring,opr_nrot,opr_violation,rings,VAdjEq,VAdjMa,b_max1len,chi0,chi0v,chi0v_C,chi0_C,chi1,chi1v,chi1v_C,chi1_C,Kier1,Kier2,Kier3,KierA1,KierA2,KierA3,KierFlex,zagreb,balabanJ,BCUT_PEOE_0,BCUT_PEOE_1,BCUT_PEOE_2,BCUT_PEOE_3,BCUT_SLOGP_0,BCUT_SLOGP_1,BCUT_SLOGP_2,BCUT_SLOGP_3,BCUT_SMR_0,BCUT_SMR_1,BCUT_SMR_2,BCUT_SMR_3,diameter,petitjean,petitjeanSC,GCUT_PEOE_0,GCUT_PEOE_1,GCUT_PEOE_2,GCUT_PEOE_3,GCUT_SLOGP_0,GCUT_SLOGP_1,GCUT_SLOGP_2,GCUT_SLOGP_3,GCUT_SMR_0,GCUT_SMR_1,GCUT_SMR_2,GCUT_SMR_3,radius,VDistEq,VDistMa,weinerPath,weinerPol,a_acc,a_acid,a_base,a_don,a_donacc,a_hyd,vsa_acc,vsa_acid,vsa_base,vsa_don,vsa_hyd,vsa_other,vsa_pol,PC+,PC-,RPC+,RPC-,Q_PC+,Q_PC-,Q_RPC+,Q_RPC-,Q_VSA_FHYD,Q_VSA_FNEG,Q_VSA_FPNEG,Q_VSA_FPOL,Q_VSA_FPOS,Q_VSA_FPPOS,Q_VSA_HYD,Q_VSA_NEG,Q_VSA_PNEG,Q_VSA_POL,Q_VSA_POS,Q_VSA_PPOS,PEOE_PC+,PEOE_PC-,PEOE_RPC+,PEOE_RPC-,PEOE_VSA+0,PEOE_VSA+1,PEOE_VSA+2,PEOE_VSA+3,PEOE_VSA+4,PEOE_VSA+5,PEOE_VSA+6,PEOE_VSA-0,PEOE_VSA-1,PEOE_VSA-2,PEOE_VSA-3,PEOE_VSA-4,PEOE_VSA-5,PEOE_VSA-6,PEOE_VSA_FHYD,PEOE_VSA_FNEG,PEOE_VSA_FPNEG,PEOE_VSA_FPOL,PEOE_VSA_FPOS,PEOE_VSA_FPPOS,PEOE_VSA_HYD,PEOE_VSA_NEG,PEOE_VSA_PNEG,PEOE_VSA_POL,PEOE_VSA_POS,PEOE_VSA_PPOS,ast_fraglike,ast_fraglike_ext,ast_violation,ast_violation_ext |
| MOE 3D descriptors | E,E_ang,E_ele,E_nb,E_oop,E_rele,E_rnb,E_rsol,E_rvdw,E_sol,E_stb,E_str,E_strain,E_tor,E_vdw,ASA,dens,glob,pmi,pmi1,pmi2,pmi3,pmiX,pmiY,pmiZ,npr1,npr2,rgyr,std_dim1,std_dim2,std_dim3,vol,VSA,vsurf_A,vsurf_CP,vsurf_CW1,vsurf_CW2,vsurf_CW3,vsurf_CW4,vsurf_CW5,vsurf_CW6,vsurf_CW7,vsurf_CW8,vsurf_D1,vsurf_D2,vsurf_D3,vsurf_D4,vsurf_D5,vsurf_D6,vsurf_D7,vsurf_D8,vsurf_DD12,vsurf_DD13,vsurf_DD23,vsurf_DW12,vsurf_DW13,vsurf_DW23,vsurf_EDmin1,vsurf_EDmin2,vsurf_EDmin3,vsurf_EWmin1,vsurf_EWmin2,vsurf_EWmin3,vsurf_G,vsurf_HB1,vsurf_HB2,vsurf_HB3,vsurf_HB4,vsurf_HB5,vsurf_HB6,vsurf_HB7,vsurf_HB8,vsurf_HL1,vsurf_HL2,vsurf_ID1,vsurf_ID2,vsurf_ID3,vsurf_ID4,vsurf_ID5,vsurf_ID6,vsurf_ID7,vsurf_ID8,vsurf_IW1,vsurf_IW2,vsurf_IW3,vsurf_IW4,vsurf_IW5,vsurf_IW6,vsurf_IW7,vsurf_IW8,vsurf_R,vsurf_S,vsurf_V,vsurf_W1,vsurf_W2,vsurf_W3,vsurf_W4,vsurf_W5,vsurf_W6,vsurf_W7,vsurf_W8,vsurf_Wp1,vsurf_Wp2,vsurf_Wp3,vsurf_Wp4,vsurf_Wp5,vsurf_Wp6,vsurf_Wp7,vsurf_Wp8,ASA+,ASA-,ASA_H,ASA_P,CASA+,CASA-,DASA,DCASA,dipole,dipoleX,dipoleY,dipoleZ,FASA+,FASA-,FASA_H,FASA_P,FCASA+,FCASA-,AM1_dipole,AM1_E,AM1_Eele,AM1_HF,AM1_HOMO,AM1_IP,AM1_LUMO |
| PipelinePilot descriptors | Molecular_Weight,ALogP,LogD,Molecular_Solubility,H_Count,C_Count,N_Count,O_Count,F_Count,P_Count,S_Count,Cl_Count,Q_Count,Num_H_Acceptors,Num_H_Donors,Num_H_Acceptors_Lipinski,Num_H_Donors_Lipinski,Num_Atoms,Num_Bonds,Num_Hydrogens,Num_ExplicitHydrogens,Num_ExplicitAtoms,Num_ExplicitBonds,Num_PositiveAtoms,Num_NegativeAtoms,Num_SpiroAtoms,Num_BridgeHeadAtoms,Num_RingBonds,Num_RotatableBonds,Num_AromaticBonds,Num_BridgeBonds,Num_RingFusionBonds,Num_Rings,Num_AromaticRings,Num_RingAssemblies,Num_Rings3,Num_Rings4,Num_Rings5,Num_Rings6,Num_Rings7,Num_Rings8,Num_Rings9Plus,Num_Chains,Num_ChainAssemblies,Num_Fragments,Num_ComplexedFragments,Num_MetalAtoms,Num_Isotopes,Num_PiBonds,Num_RGroupFragments,Num_StereoAtoms,Num_StereoBonds,Num_SingleBonds,Num_DoubleBonds,Num_TripleBonds,Num_AliphaticSingleBonds,Num_AliphaticDoubleBonds,Num_UnknownStereoAtoms,Num_UnknownStereoBonds,Num_DativeBonds,Num_HydrogenBonds,Num_AlleneStereoCenters,Num_AtropisomerCenters,Num_AxialStereoCenters,Num_TrueAlleneStereoCenters,Num_TrueAtropisomerCenters,Num_AtomClasses,Num_Macro_Chains,Num_Macro_Residues,Num_TerminalRotomers,Num_TrueStereoAtoms,Num_UnknownTrueStereoAtoms,Num_PseudoStereoAtoms,Num_UnknownPseudoStereoAtoms,Num_MesoStereoAtoms,Num_EnhancedStereoAtoms,Num_QueryAtoms,Num_QueryBonds,Num_LinkAtoms,QED,Minimized_Energy,Molecular_Volume,Molecular_SurfaceArea,Molecular_PolarSurfaceArea,Molecular_FractionalPolarSurfaceArea,Molecular_SASA,Molecular_PolarSASA,Molecular_FractionalPolarSASA,Molecular_SAVol,Kappa_1,Kappa_2,Kappa_3,Kappa_1_AM,Kappa_2_AM,Kappa_3_AM,PHI,apol,max_pKa |

**Table S2.** List of 72 molecular descriptors used in the SVM model selected from the NSGA-II results.

| Descriptor | Description |
| --- | --- |
| AM1_HOMO | The energy of the Highest Occupied Molecular Orbital calculated using the AM1 Hamiltonian. |
| pKa (most basic) | The negative base-10 logarithm of the acid dissociation constant (K_a_) of a solution. |
| b_double | Number of double bonds. Aromatic bonds are not considered to be double bonds. |
| Molecular_Fractional PolarSurfaceArea | The ratio of the polar surface area divided by the total surface area. |
| SMR_VSA | Sum of the van der Waals surface area of atom i with specific range of the molecular refractivity. (8 descriptors) |
| AM1_dipole | The dipole moment calculated using the AM1 Hamiltonian |
| Num_RingFusion Bonds | Number of bonds shared by fused ring |
| vsa_acc | Approximation to the sum of VDW surface areas (Å^2^) of pure hydrogen bond acceptors. |
| logS | Log of the aqueous solubility. |
| logP(o/w) | Log of the octanol/water partition coefficient. |
| vsurf_CW | Capacity factor. (8 descriptors) |
| PC_plus | Total positive partial charge: the sum of the positive qi. Q_PC+ is identical to PC+, which has been retained for compatibility. |
| Num_DoubleBonds | Number of double bonds. |
| PEOE_VSA_plus | Sum of the van der Waals surface area of atom i with specific range of the partial charge. (7 descriptors) |
| vsurf_A | Amphiphilic moment. |
| vsurf_R | Surface rugosity. |
| a_don | Number of hydrogen bond donor atoms. |
| Num_H_Acceptors | Number of hydrogen bond acceptor atoms. |
| a_aro | Number of aromatic atoms. |
| vsa_don | Approximation to the sum of VDW surface areas of pure hydrogen bond donors. |
| GCUTS_SMR | The GCUT descriptors* using atomic contributions to molar refractivity using the Wildman and Crippen SMR method. (4 descriptors) |
| SlogP_VSA | Sum of the van der Waals surface area of atom i with specific range of SlogP. (10 descriptors) |
| a_acc | Number of hydrogen bond acceptor atoms (not counting acidic atoms but counting atoms that are both hydrogen bond donors and acceptors. |
| a_acid | Number of acidic atoms. |
| vsa_base | Approximation to the sum of VDW surface areas of basic atoms. |
| opr_brigid | The number of rigid bonds from Oprea 2000. |
| AM1_HF | The heat of formation (kcal/mol) calculated using the AM1 Hamiltonian. |
| AM1_LUMO | The energy (eV) of the Highest Occupied Molecular Orbital calculated using the AM1 Hamiltonian. |
| GCUT_PEOE | The GCUT descriptors using atomic contribution to PEOE partial charges.(4 descriptors) |
| Minimized_Energy | The energy after the minimization procedure. |
| Num_Aliphatic SingleBonds | Number of single bonds between heavy atoms that are not in aromatic rings. |
| Num_ChainAssemblies | Chain assemblies, defined as the fragments remaining when all ring bonds are removed from the molecule. |
| Num_AtomClasses | Different atom classes from symmetry perception (excluding hydrogens). |
| Num_RingAssemblies | Ring assemblies, defined as the fragments remaining when all non-ring bonds are removed from the molecule. |
| Num_Rings6 | Number of rings of size 6. |
| Num_Rings7 | Number of rings of size 7. |
| Num_TerminalRotomers | A terminal rotomer is defined as either a non-terminal sp^3^ atom connected to three terminal atoms of the same type, or a non-terminal sp^2^ atom connected to two terminal atoms of the same type. |

* GCUT descriptors are calculated from the eigenvalues of a modified graph distance adjacency matrix.

**Table S3.** The Kappa statistics and the ROC scores of the SVM models using (1) the 72 descriptors selected by NSGA-II and ECFP_4, and (b) ECFP_4, for all the combinations between the training set and the test set derived from each database.

(a)

|  |  | Test set | | | | |
| --- | --- | --- | --- | --- | --- | --- |
|  |  | ChEMBL | GOSTAR | NCGC | hERG  Central | Integrated  dataset |
|  |  | Kappa ROC | Kappa ROC | Kappa ROC | Kappa ROC | Kappa ROC |
| Training set | ChEMBL | 0.769 0.946 | 0.593 0.870 | 0.602 0.881 | 0.086 0.733 | 0.459 0.858 |
|  | GOSTAR | 0.572 0.857 | 0.763 0.959 | 0.451 0.828 | 0.027 0.640 | 0.140 0.774 |
|  | NCGC | 0.441 0.811 | 0.456 0.760 | 0.595 0.851 | 0.060 0.717 | 0.246 0.809 |
|  | hERG  Central | 0.020 0.732 | 0.104 0.675 | 0.218 0.810 | 0.547 0.929 | 0.321 0.923 |
|  | Integrated  dataset | 0.740 0.943 | 0.755 0.954 | 0.575 0.872 | 0.551 0.930 | 0.729 0.962 |

(b)

|  |  | Test set | | | | |
| --- | --- | --- | --- | --- | --- | --- |
|  |  | ChEMBL | GOSTAR | NCGC | hERG  Central | Integrated  dataset |
|  |  | Kappa ROC | Kappa ROC | Kappa ROC | Kappa ROC | Kappa ROC |
| Training set | ChEMBL | 0.585 0.870 | 0.218 0.691 | 0.386 0.831 | 0.063 0.731 | 0.199 0.814 |
|  | GOSTAR | 0.182 0.652 | 0.594 0.906 | 0.192 0.683 | 0.015 0.625 | 0.033 0.624 |
|  | NCGC | 0.066 0.627 | 0.087 0.564 | 0.311 0.791 | 0.053 0.643 | 0.078 0.677 |
|  | hERG  Central | 0.019 0.638 | 0.073 0.567 | 0.157 0.799 | 0.494 0.914 | 0.283 0.878 |
|  | Integrated  dataset | 0.554 0.864 | 0.578 0.88 | 0.305 0.846 | 0.509 0.912 | 0.642 0.947 |

**Table S4.** Prediction performance of the SVM-model for data sets within and outside of the applicability domain defined by a similarity threshold of 0.6 (inside: ≥ 0.6, outside: < 0.6).

| Applicability domain | TP/FN  FP/TN | Accuracy | Sensitivity | Specificity | BAC | Kappa |
| --- | --- | --- | --- | --- | --- | --- |
| Outside | 163/253  44/12,059 | 0.976 | 0.392 | 0.996 | 0.694 | 0.512 |
| Inside | 1,824/726  366/71,926 | 0.985 | 0.715 | 0.995 | 0.855 | 0.762 |
